# An Environmentally-Friendly RNAi Yeast-Attractive Targeted Sugar Bait Turns off the *Drosophila suzukii Rbfox1* Gene

**DOI:** 10.3390/insects16050481

**Published:** 2025-05-01

**Authors:** Keshava Mysore, Jackson Graham, Saisuhas Nelaturi, Teresia M. Njoroge, Majidah Hamid-Adiamoh, Akilah T. M. Stewart, Longhua Sun, Molly Duman-Scheel

**Affiliations:** 1 Department of Medical and Molecular Genetics, Indiana University School of Medicine, Raclin-Carmichael Hall, 1234 Notre Dame Ave., South Bend, IN 46617, USA; kmysore@iu.edu (K.M.); tenjorog@iu.edu (T.M.N.); mhamidad@iu.edu (M.H.-A.); akilstew@iu.edu (A.T.M.S.); lsun@nd.edu (L.S.); 2 Eck Institute for Global Health, The University of Notre Dame, Notre Dame, IN 46556, USA; jgraham9@nd.edu (J.G.); snelatur@nd.edu (S.N.); 3 Department of Biological Sciences, The University of Notre Dame, Notre Dame, IN 46556, USA; 4 Department of Chemistry and Biochemistry, The University of Notre Dame, Notre Dame, IN 46556, USA

**Keywords:** berry, control, eco-friendly, fruit crop, integrated pest management, insect, insecticide, spotted wing drosophila

## Abstract

Spotted wing drosophila (SWD), *Drosophila suzukii* (Diptera: Drosophilidae), are invasive vinegar flies of East Asian origin that are an increasingly global threat to the small fruit industry. It is essential that new classes of eco-friendly insecticides and cost-effective strategies for SWD control are developed. Here, we describe the preparation of a strain of RNA interference (RNAi) *Saccharomyces cerevisiae* expressing shRNA that specifically targets the SWD *RNA-binding Fox protein 1 (Rbfox1)* gene. The yeast effectively silences the SWD *Rbfox1* gene, resulting in significant loss of fly neural activity. Laboratory trials demonstrated that the RNAi yeast can be mixed with soda, which functions as SWD attractive targeted sugar bait (ATSB) that can be delivered in a soda bottle feeder. The ATSB, mixed with yeast that was heat-killed prior to suspension in the ATSB, resulted in 92 ± 1% mortality of SWD flies that consumed it, yet had no impact on non-target dipterans. Rbfox.687 yeast delivered in ATSB feeders may one day be a useful component of integrated SWD control programs.

## Simple Summary

It is critical that we identify new eco-friendly methods of controlling spotted wing drosophila (SWD), a destructive fruit and berry crop pest. This study evaluated RNAi insecticides that specifically turned off an essential SWD gene. The RNAi insecticides were produced and delivered to flies in baker’s yeast that was mixed with soda. The yeast effectively killed SWD, but did not impact other insects. These insecticides may represent a new tool for controlling SWD.

## 1. Introduction

*Drosophila suzukii* are vinegar flies of East Asian origin that have wreaked havoc on the small fruit industry worldwide [1,2]. Modeling predicts that *D. suzukii* will likely be broadly present in the United States, southeast Asia, Australia, and Europe, with considerable expansion of its range in the northern hemisphere [3]. SWD impact most berry crops, cherries, grapes, and other tree fruits [4]. The flies oviposit within ripe fruits, where the larvae hatch and compromise fruit integrity [1]. SWD complete multiple generations in a single year [1]. In locations where SWD are well established, weekly insecticide applications are necessary. These weekly applications result in increased costs and unwanted harm to non-targeted organisms [5]. Calendar spray programs that use broad-spectrum insecticides, which employ organophosphates and pyrethroids, are common among berry farmers [6,7]. With increased insecticide treatments, it was recently demonstrated that there is potential for the emergence of resistance to organophosphates and pyrethroids in this species [8,9,10]. It is therefore critical to identify new classes of pesticides and technologies for controlling SWD.

RNAi is often used to characterize gene function in the laboratory. Although it has generated interest in the insect control realm, few have successfully translated it from the bench to the field [11]. RNAi pesticides targeting neural genes in multiple species of vector mosquitoes were recently generated [12,13]. These mosquito-specific RNAi insecticides were designed to target nucleotide stretches conserved in mosquitoes but not in any other organisms, including humans. For example, an RNAi yeast insecticide was designed to target a conserved site in the mosquito *Rbfox1* gene [13]. *Rbfox1* genes, which are also referred to as *Ataxin 2-binding protein* genes, encode an evolutionarily conserved RNA-binding protein that functions in many different biological processes. Rbfox1 protein binds to UGCAUG motifs located in pre-mRNA introns, 3′ UTRS, and pre-miRNA hairpins; this enables the regulation of splicing, mRNA stability, translation, and miRNA processing by Rbfox1 proteins [14,15]. Rbfox1 also promotes ribonucleoprotein granule formation and the survival of cells [16]. In the mosquito *Aedes aegypti,* silencing of the *Rbfox1* gene resulted in high levels of mosquito mortality. The mortality correlated with severe defects in neural activity within the mosquito brain, in which Rbfox was shown to be a positive regulator of *Notch* signaling. The insecticidal impacts of *Rbfox1* silencing were subsequently confirmed in trials conducted on additional human disease vector species, including *Aedes albopictus*, *Anopheles gambiae*, and *Culex quinquefasciatus* [13].

The RNAi insecticides characterized in mosquitoes consisted of short hairpin RNA (shRNA) molecules that were expressed in baker’s yeast, *S. cerevisiae*, which is consumed by mosquitoes, resulting in silencing of the target neural genes [12,13]. The use of *S. cerevisiae* enables inexpensive and scalable preparation of the insecticides, and the insecticidal activity of the yeast is maintained when it is heat-killed and dried [17]. Moreover, the yeast can be delivered to mosquitoes as the active ingredient in ATSBs [18], which are presently being assessed for the control of vector insect pests [19,20,21,22]. These insecticidal baits capitalize on the natural sugar-feeding behavior of insects that are attracted to feed on a sugar source laced with a poison [23]. In recent studies, ATSBs targeting mosquitoes were mixed with RNAi yeast [24]. Characterization of the RNAi yeast ATSBs against mosquitoes in both laboratory and semi-field settings has demonstrated the efficacy and biorationality of these pesticides [24]. Several of the targeted genes, which function in the nervous system [24], have orthologues in *D. suzukii*. Recent studies have shown that baker’s yeast, *S. cerevisiae,* is an effective attractant of major agricultural pests [25,26]. Moreover, sucrose is known to improve insecticide activity against *D. suzukii* [27]. Combined, these findings support the hypothesis that species-specific RNAi yeast ATSBs targeting SWD can be generated. Here, we describe the development and characterization of an RNAi yeast ATSB targeting the SWD *Rbfox1* gene.

## 2. Materials and Methods

### 2.1. Insect Rearing

A *D. suzukii* strain established from a local Michigan collection was obtained from Juliana Wilson (Michigan State University, East Lansing, MI, USA). The flies were reared at 26 °C and maintained in bottles containing Nutri-Fly^®^ BF media (Genesee Scientific, El Cajon, CA, USA).

### 2.2. Yeast Engineering and Culturing

An RNAi yeast strain expressing shRNA targeting the *D. suzukii Rbfox1* gene (NCBI reference sequence: XR_010654136) target site 5′-CCATTGGCGATACTATCCAATCCGG-3′ was prepared as previously described [17]. The *S. cerevisiae CEN.PK* yeast strain (genotype *MATa/α ura3-52/ura3-52 trp1-289/trp1-289 leu2-3_112/leu2-3_112 his3 Δ1/his3 Δ1 MAL2-8C/MAL2-8C SUC2/SUC2*) [28] was transformed with a pRS426GPD plasmid [29] containing the *Rbfox1* shRNA expression cassette that was prepared as described [30] and selected through growth on a media-lacking uracil. This strain will hereafter be referred to as Rbfox.687. A control yeast strain expressing shRNA with no known target in SWD [17] was also prepared for control studies. Yeast was cultured and heat-killed upon harvesting as described [17] and then lyophilized with 0.025% benzoic acid, which was added as a preservative.

### 2.3. RNAi Sugar Feeding Assays

*Petri dish assays:* 3–4 day-old flies were transferred into an empty bottle and starved for 4–5 h. Following the starvation period, the bottle of flies was placed on ice for 15–20 min. A total of 100 µL of a sugar bait–yeast mixture was prepared by mixing 10% sucrose solution (ASB) with 4.5% red food coloring and 40 mg of control RNAi or treatment yeast using a sterile toothpick. Four ~25 µL drops of the ASB + yeast mixture were placed on a Petri dish. Following anesthetization on ice, 25 flies (a mixture of males and females) were placed in the Petri dish, which was subsequently covered with a lid. Flies were permitted to feed overnight in the RNAi yeast–sugar feeding assay, which was conducted at room temperature (~21 ± 1 °C). Feeding was verified in flies by confirming the presence of red food dye in the fly abdomens. Following the overnight feeding, flies were again placed on ice during transfer to a fresh food bottle, in which behavioral phenotypes and survival were evaluated for the next six days.

*Dose–response curves:* Dose–response assays were performed as previously described [17] by assessing different concentrations of Rbfox.687, which were prepared with varying amounts of control and Rbfox.687 yeast. Each concentration of Rbfox.687 was tested on 25 individual flies per treatment in a Petri dish, which had been prepared as described above. Microsoft Excel 365 software was used to graph the data, which were evaluated through probit analyses conducted using SPSS 25 software (IBM, Armonk, NY, USA).

*Yeast endless soda (YES) feeder assays:* A feeder system was prepared as described [31] using MUDUODUO automatic bird drinker cups (Amazon, Seattle, WA, USA) that were modified to create the ATSB feeders. A small piece of dehumidifier filter (Honeywell Home, Charlotte, NC, USA) lacking the metal layer was placed in the channel between the reservoir and feeding area. The channel as well as the reservoir were wrapped with parafilm to prevent leakage. The parafilm also prevented the escape of flies into the reservoir and subsequent drowning. To prepare the feeder side, pieces of filter were cut into a 3.3 cm × 7 cm rectangle and folded into a circle. 5 µm (bottom) and 90 µm (top) nylon membranes were placed on top. 200 mg of either control or treatment yeast was mixed with 350 µL of degassed/flat Coca-Cola^R^, creating a paste that was placed between the membrane layers. At the other end of the feeder, a 12 fl oz soda bottle containing 110 mL of degassed temperature-equilibrated Coca-Cola^R^ and 10 mL/L Tegosept anti-mold agent (Thermofisher Scientific, Waltham, MA, USA) was inverted to serve as a continuous supply of soda sugar bait. For feeding assays, the feeder and 50 3–4 day-old sugar-starved flies were placed in insect cages that were placed in the insectary, where they were monitored for six days, with mortality recorded daily. These data were tabulated and graphed using Microsoft Excel 365 software and evaluated using one-way ANOVA statistics and a Tukey’s post hoc test performed with SPSS 25 (IBM, Armonk, NY, USA).

### 2.4. Confirmation of Rbfox1 Silencing

*Rbfox1* target gene silencing was confirmed through qRT-PCR assays that were performed as described [32]. Fly heads were collected 72 h after ATSB feedings (control or treatment). Total head RNA was extracted from 20 females using the Trizol reagent (Invitrogen, Carlsbad, CA, USA) according to the manufacturer’s instructions. The RNA was subsequently treated with DNase I (using the DNA-free kit, Invitrogen, Thermo Fisher Scientific, Waltham, MA, USA) per the manufacturer’s instructions. cDNA was prepared from RNA using the HighCapacity RNA to cDNA Kit (Applied Biosystems, Foster City, CA, USA) according to the manufacturer’s instructions. Real-time PCR quantification assays were performed using a CFX Opus 96 Real-time PCR System (Bio-Rad) using the Power SYBR Green PCR Master Mix as described by the manufacturer (Bio-Rad, Hercules, CA, USA). To amplify the *Rbfox1* gene, the following primer sets were used in these reactions: forward 5′-CCCACCACCGGATTGGATAG-3′ and reverse: 5′-GCGGAACGGTATGTTGGAGA-3′. Amplification of *alpha tubulin*, an internal standard for data normalization, was performed as described [32] with the following primers: forward 5′-AGGATGCGGCGAATAACT-3′ and reverse 5′ CGGTGGATAGTCGCTCAA 3′. PCR amplifications were performed in six replicate wells in each of two separate biological replicate trials, and results were quantified by standardizing reactions to *alpha tubulin* transcript levels using the ΔΔCt method as described [32]. Data were statistically evaluated using Student’s *t*-test.

### 2.5. Evaluation of Yeast Toxicity to Non-Target Insects

The effects of Rbfox.687 yeast feedings on non-target insects were analyzed in *Aedes aegypti* (Liverpool-IB12)*, Anopheles stephensi* STE2 (obtained through BEI Resources, Manassas, VA, USA, NIAID, NIH: *A. stephensi* strain STE2, MRA-128, contributed by William E. Collins)*, Culex quinquefasciatus* JHB (provided by the Centers for Disease Control and Prevention for distribution by BEI Resources, Manassas, VA, USA, NIAID, NIH: Eggs, NR-43025), *Drosophila melanogaster,* and *Pogonomyrmex barbatus* (Carolina Biologicals, Burlington, NC, USA). These insects were selected because cultures were concurrently being maintained in the lab during the course of the SWD study. In these trials, yeast–sugar feeding assays were performed as previously described [13]. The same preparation of yeast was used for all trials. For a positive control, the same yeast was shown to kill SWD in concurrent assays.

### 2.6. Immunohistochemistry Studies

The following reagents were used for immunohistochemical staining of adult *D. suzukii* brains, which were performed as previously described [33,34]: anti-HRP (Jackson ImmunoResearch Labs, West Grove, PA, USA), mAb nc82 anti-Bruchpilot [35] (DSHB, Iowa City, IA, USA, Iowa Hybridoma Product nc82 deposited by E. Buchner), and TO-PRO-3 iodide (Molecular Probes, Eugene, OR, USA). Immunohistochemical assays were performed in triplicate, and processed tissues were mounted and imaged with a Zeiss 710 confocal microscope. Confocal images of the fly brains were analyzed with FIJI ImageJ version 2.16.0/1.54p, [36] and Adobe Photoshop 2025 software. For quantification of signal intensities, mean gray values (average signal intensity over the selected area) were calculated as described [12] and statistically analyzed using a Student’s *t*-test.

## 3. Results and Discussion

### 3.1. SWD-Specific Insecticidal Activity of Rbfox.687 Yeast

**Figure 1:**
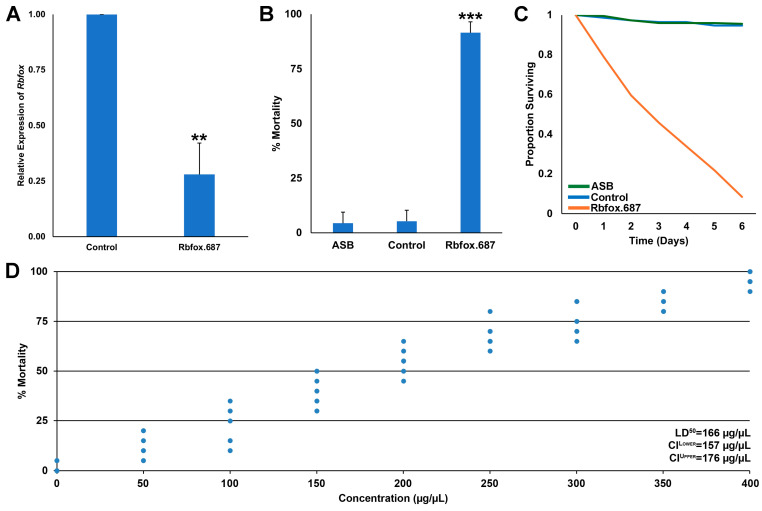
Rbfox.687 yeast functions as the active ingredient in an ATSB that silences the *Rbfox1* gene and kills SWD. (**A**) qRT-PCR confirmed that the SWD *Rbfox1* gene was silenced in the brains of adult flies that consumed Rbfox.687 yeast (** = *p* < 0.01 vs. control, Student’s *t*-test). (**B**) Laboratory trials demonstrated that consumption of Rbfox.687 yeast resulted in significant mortality of SWD flies (*** = *p* < 0.001 vs. control, Student’s *t*-test). (**C**) The corresponding survival curve for these data are shown. Panels (**B**,**C**) were compiled from nine replicate trials for each treatment, each of which contained 25 adults. The error bars represent the standard deviation (SD) in (**A**) and the standard error of the mean (SEM) in (**B**). (**D**) Dose-dependent mortality was observed in *D. suzukii*, with an LD_50_ of 166 µg/µL; the data shown were compiled from 10 replicate trials (each with 25 flies) for each of the nine different concentrations of yeast.

A recent study demonstrated that consumption of yeast targeting a site conserved in mosquito *Rbfox1* genes resulted in loss of *Rbfox1* transcripts and mosquito mortality [13]. It was therefore hypothesized that RNAi yeast Rbfox.687, which was designed to specifically target a 25 bp sequence present in the *D. suzukii Rbfox1* gene, would silence the *Rbfox1* gene and lead to fly death. To test the hypothesis, heat-inactivated dried Rbfox.687 yeast was mixed with sucrose and fed to SWD in sugar feeding assays conducted in Petri dishes. As predicted, 72% silencing of *Rbfox1* transcripts was observed in the brains of flies that had consumed Rbfox.687 yeast (Figure 1A, *p* < 0.001 vs. control yeast-treated flies). Significant SWD mortality was observed in flies that had consumed Rbfox.687 (Figure 1B, *p* < 0.001 vs. sugar bait only or control yeast-treated flies, which survived), with death observed over a six day period (Figure 1C). Dose–response assays revealed an LD_50_ of 166 μg/μL for Rbfox.687 yeast (Figure 1D). The high levels of SWD mortality observed suggest that the yeast-mediated delivery of insecticidal shRNA can overcome the activity of dsRNAses that are present in the *D. suzukii* gut, which are known to reduce RNAi efficacy in SWD [37]. These results suggest that RNAi yeast insecticides may prove to have higher field efficacy than other RNAi insecticides.

RNAi insecticides, including Rbfox.687, that use shRNA as the interfering RNA species facilitate precision pest control. For example, the Rbfox insecticide trialed in mosquitoes was designed to match a 25 bp target site conserved in a variety of vector mosquitoes but not found in other genomes; the insecticide killed *A. aegypti*, *A. gambiae*, and *C. quinquefasciatus*, but it did not kill non-targeted insect species [13]. Similarly, the Rbfox.687 yeast was designed to target a short 25 bp site found in *D. suzukii* but which is not present in mosquitoes and other organisms. NCBI Blast searches failed to detect identical 25 bp target sites in other sequenced genomes. Consequently, although Rbfox.687 kills *D. suzukii*, sugar feeding trials demonstrated that the yeast did not kill *A. aegypti*, *A. stephensi*, *C. quinquefasciatus*, *D. melanogaster*, or *P. barbatus* that consumed Rbfox.687 yeast (*p* > 0.05 vs. control-yeast treated SWD, Table 1). This lack of toxicity to non-target species, which is enhanced by the use of a very short 25 bp target sequence, would support future regulatory applications. Furthermore, the use of heat-killed dried yeast distinguishes this yeast technology from other systems previously developed for *D. suzukii*, which used live yeast and targeted long stretches of RNA [38]. This distinction can enable classification of the Rbfox.687 insecticide as a dead microbe. The species-specificity of the dead microbial insecticide is likely to facilitate registry of RNAi yeast insecticides with regulatory authorities.

### 3.2. Silencing of Rbfox1 Results in Loss of Neural Activity in the Brain and Behavioral Phenotypes in Adult Flies

**Figure 2:**
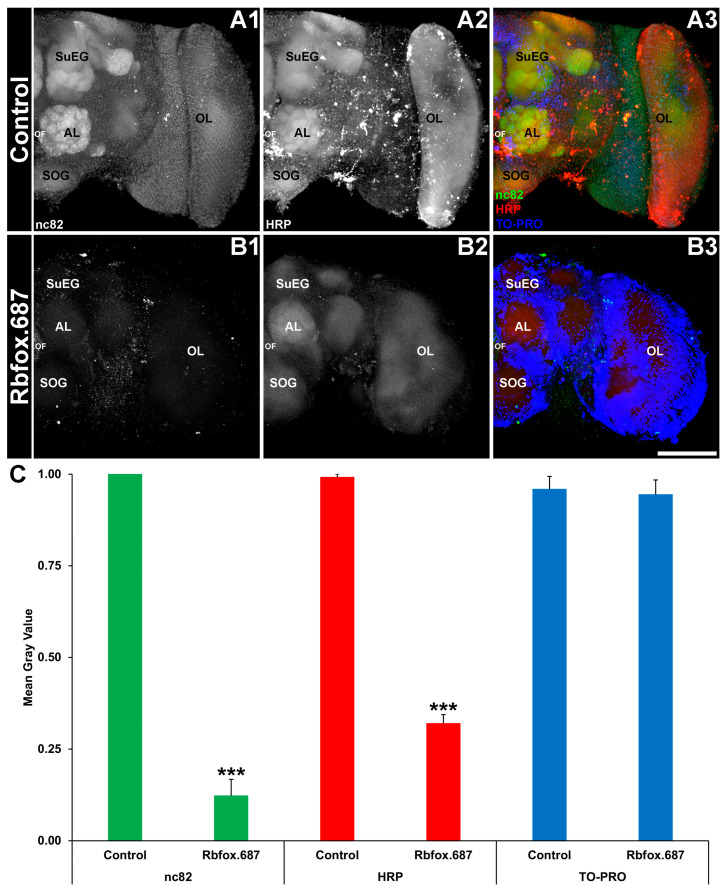
Neural defects are detected in SWD that consumed Rbfox.687 yeast ATSB. Adult brains prepared from flies that consumed control (**A1**–**A3**) or Rbfox.687 (**B1**–**B3**) yeast were labeled with mAbnc82 (marker for active synapses; white in (**A1**,**B1**), green in (**A3**,**B3**)), anti-HRP (neural marker; white in (**A2**,**B2**), red in (**A3**,**B3**)) and TO-PRO (nuclear stain; blue in (**A3**,**B3**)). Although nc82 and HRP levels were significantly reduced in Rbfox.687-treated brains (**C**); *** *p* < 0.001 vs. control), no significant levels of nuclear staining were detected (**C**, *p* > 0.05). The data in panel (**C**) are shown as average mean gray values with error bars denoting the SEM and were analyzed with Student’s *t*-test. Representative larval brains are oriented dorsal upward and labeled as follows: AL, larval antennal lobe; OF, esophageal foramen; OL, optic lobe; SOG, sub-esophageal ganglion; and SuEG, supraesophageal ganglion. Scale Bar = 100 μm. N = 75 brains/treatment from three replicate trials.

In adult mosquitoes, consumption of yeast corresponding to the *Rbfox1* gene resulted in loss of neural activity in the adult brain [13]. Based on these results, it was hypothesized that silencing of *Rbfox1* in *D. suzukii* would result in comparable neural defects. Treatments with Rbfox.687 yeast resulted in a reduction in nc82 levels in the adult SWD brain (Figure 2A1 vs. Figure 2B1; green), which correlates well with the loss of the *Rbfox1* transcript observed in the fly head (Figure 1A). Although levels of Bruchpilot, a marker of active synapses [35] that are labeled with the nc82 antibody, as well as HRP levels (Figure 2A2 vs. Figure 2B2) were significantly reduced in flies that consumed the insecticidal yeast, no significant difference in TO-PRO nuclear staining levels (Figure 2A3 vs. Figure 2B3) was observed in the fly brain (Figure 2C; *p* > 0.05). These results, which were similar to those observed in *A. aegypti* [13], suggested that loss of nc82 signal likely results from a reduction in neural activity rather than loss of neural density.

The silencing of SWD *Rbfox 1* (Figure 1A) and neural deficits (Figure 2) correlated with a locomotor defect and the inability to fly. Although flies can survive for up to six days following yeast treatment in the laboratory, it is likely that they would die sooner in field assays in which they were susceptible to predation or exposed to outdoor elements. This was the case for mosquitoes that had locomotor defects resulting from *Shaker* gene silencing, which died within one day following yeast treatment in semi-field experiments conducted outdoors in Trinidad and in Thailand [24].

### 3.3. An ATSB Station for Delivery of Insecticidal RNAi Yeast

**Figure 3:**
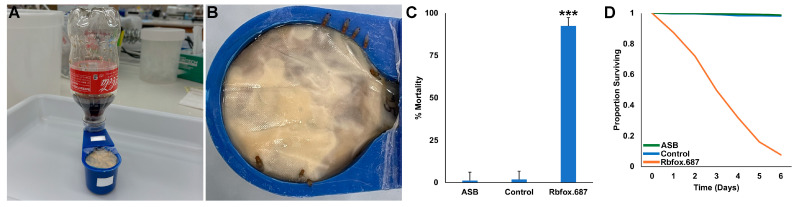
Rbfox.687 yeast and soda delivered in a soda bottle feeder induced significant SWD mortality. (**A**) The soda bottle feeder system is shown. (**B**) SWD feeding on the yeast-soda mixture were observed. (**C**) Significant mortality was observed in *D. suzukii* that drank from a soda bottle ATSB feeder prepared with Coca-Cola^R^ and Rbfox.687 yeast (*** *p* < 0.001 vs. flies that fed on soda alone or soda prepared with control yeast, ANOVA). (**D**) The flies died over a six day period. Data compiled from nine replicate trials for each treatment, each with 50 adults are shown in (**C**,**D**).

The use of RNAi yeast insecticides for SWD control would require the development of means for delivering the insecticides in the field. Coca-cola^R^ is known to be an excellent sugar bait attractant for *Aedes japonicus* [39], and we have recently begun to explore the use of Coca-Cola^R^ as a sugar bait in a variety of insects, including SWD [31]. Recent laboratory trials demonstrated that yeast could be delivered to insects in a feeder composed of a soda bottle attached to a reservoir containing yeast, which had been treated with a mold inhibitor [31]. The bottle feeder, which is shown in Figure 3A, was constructed so that the yeast, which was covered by a membrane, was constantly rewetted, thereby preventing yeast drying. Rbfox.687 yeast was mixed with Coca-Cola^R^ and supplied to SWD in the soda bottle feeders that continuously rewetted the yeast, which was consumed by the flies through a membrane (Figure 3B). Rbfox.687 yeast delivered to SWD in this manner resulted in significant SWD mortality (92 ± 1%, Figure 3C,D, *p* < 0.001 vs. soda only or soda + control yeast-treated flies).

The results of these simulated field soda bottle feeder assays are promising. It will be important to next investigate the efficacy of RNAi yeast soda and bottle feeders in the field. The results of such trials will be critical for determining when and where to place the feeders and whether the soda–yeast system can effectively compete against natural SWD sugar sources. During the cold season in temperate zones, the adult population is composed primarily of flies that are active during daytime hours, when temperature permits, and reside within areas containing wild vegetation [40]. Although the source of food during the cold season is presently unknown for SWD, it is likely that sugar source availability is restricted. It may therefore be useful to deploy ATSB feeders toward the end of the cold season, when temperatures are warmer, but before fruit crops are available. Recent studies have demonstrated that winter morphs, which are thought to be the main source for infestations of fruit crops [41], are highly attracted to yeast, which could be beneficial [42]. Insecticide sprays are frequently used for SWD control [40], suggesting that the development of RNAi yeast spray formulations might also be of interest. Such sprays have been developed for bacterial larvicides [43]. SWD primarily come into contact with insecticides sprayed on fruits and foliage where they feed, oviposit, and develop to maturity [40], so it would be important to evaluate the best areas to deploy RNAi yeast spray insecticides if such sprays were to be developed.

If the efficacy of RNAi yeast insecticides is demonstrated, the use of this technology could benefit SWD integrated pest management (IPM) programs, which presently rely heavily on the use of chemical insecticides [6,40]. The introduction of RNAi yeast-dead microbial pesticides to the current repertoire could potentially help reduce chemical inputs while providing an SWD-specific pesticide that would be safer for non-target organisms. Moreover, this dead microbial pesticide could potentially be of interest to organic fruit farmers, who have been particularly hard hit by the invasion of SWD due to the limited ability of effective insecticides that can be used to protect organic crops [40].

The cost of RNAi insecticides will of course also be a critical factor [40]. The use of yeast is expected to significantly reduce the cost of RNAi-based interventions. Recent studies demonstrated that commercial-ready RNAi yeast could be produced at pilot scale with no indication that special media, which increase prices substantially, would be necessary, suggesting that the yeast could be produced at competitive prices [44]. The Rbfox.687 laboratory yeast strain used in the present study is not suitable for scaled fermentation. For scaling, it would be useful to integrate multiple copies of the Rbfox.687 shRNA expression construct into a commercial yeast strain. Cas-CLOVER was recently used to produce such yeast strains, in which shRNA production levels increased ~30 fold in pilot scaled fermentations [44].

Inexpensive scaled production of insecticidal yeast would facilitate further evaluation of RNAi yeast efficacy when deployed in YES feeders. In a recent study [45], ATSB stations deployed on household structures for mosquito control in Western Zambia were often damaged. These findings underline the importance of assessing whether the YES feeders or other potential deployment systems are suitable for use in agricultural settings and how often the feeders might need to be replaced. Potential agricultural stakeholders should also have the opportunity to voice any concerns regarding the use of this novel technology and to make any recommendations for improvement. When community acceptance of the ATSB stations for mosquito control was evaluated in Zambia, stakeholders accepted the novel mosquito control intervention [46]. The demonstration of similar levels of acceptance of the RNAi yeast/YES feeders in conjunction with significantly reduced SWD densities in multiple agricultural settings would be ideal. ATSB stations significantly reduced the incidence of malaria mosquito vectors in Mali [21,22], suggesting that similarly reduced densities of SWD might be achieved. The ATSB used in Mali was shown to effectively compete with natural nectar sources [47], suggesting that the intervention has the capacity to reduce mosquito densities following deployment. In a related mosquito ATSB study conducted in Western Zambia, ATSB feeding rates in *Anopheles funestus* mosquitoes were consistent with those expected to reduce malaria transmission; however, vector light trapping densities were not significantly reduced [48]. Consistent with these findings, the deployment of two ATSB stations per household in rural Western Zambia did not result in a statistically significant reduction in clinical malaria incidences among children [20]. The authors concluded that further research is needed to optimize the impact of ATSB deployment for vector control in Zambia and other settings [20]. Thus, future research endeavors to assess the efficacy of RNAi yeast/YES feeders for SWD control should be performed in multiple agricultural contexts in various crop and climate settings.

## 4. Conclusions

The results of these studies indicate that Rbfox.687, a species-specific RNAi yeast insecticide that targets *D. suzukii,* can serve as a highly toxic component of an ATSB that effectively kills SWD, yet has no impact on non-target insects. Rbfox.687 yeast ATSB silences the *D. suzukii Rbfox1* gene, resulting in neural defects in the adult SWD brain. The Rbfox.687 yeast was delivered in a soda bottle feeder that effectively killed *D. suzukii* under simulated field conditions. RNAi yeast insecticides represent a new class of effective, species-specific biorational insecticides that could one day become an important component of integrated pest management programs for SWD control following field studies designed to optimize the impacts of RNAi yeast-ATSB deployment in a variety of agricultural contexts.

## Figures and Tables

**Table 1:** Rbfox.687 yeast is not toxic to non-target arthropods. Consumption of Rbfox.687 yeast by the indicated non-target insects had no significant impact on insect survival (*p* > 0.05, Student’s *t*-test).

Test Organism	% Survival ± SD		
	n/Treatment	Control Yeast	Rbfox.687
*P. barbatus*	50	95 ± 3	95 ± 5
*A. aegypti*	50	98 ± 2	99 ± 1
*A. stephensi*	50	100 ± 1	99 ± 1
*C. quinquefasciatus*	50	100 ± 0.4	100 ± 0
*D. melanogaster*	50	100 ± 1	99 ± 1

## Data Availability

The original contributions presented in this study are included in the article. Further inquiries can be directed to the corresponding author.

## Abbreviations

The following abbreviations are used in this manuscript:

ATSB	Attractive targeted sugar bait
IPM	Integrated pest management
RNAi	RNA interference
*Rbfox1*	*RNA Binding Fox-1 Homolog*
SD	Standard deviation
SEM	Standard error of the mean
shRNA	Short hairpin RNA
SWD	Spotted-wing drosophila
YES	Yeast endless soda
